# An Assessment of the Knowledge and Attitudes of Final-Year Dental Students on and Towards Antibiotic Use: A Questionnaire Study

**DOI:** 10.3390/antibiotics14070645

**Published:** 2025-06-25

**Authors:** Ozgun Yildirim, Humeyra Yildiz, Nur Mollaoglu

**Affiliations:** 1 Department of Oral and Maxillofacial Surgery, Gülhane School of Dentistry, Health Sciences University, 06010 Ankara, Turkey; 2 Department of Oral and Maxillofacial Surgery, School of Dentistry, Gazi University, 06490 Ankara, Turkey; humeyra.yildiz@gazi.edu.tr (H.Y.); nurmolla@gazi.edu.tr (N.M.)

**Keywords:** antibiotic, dental treatment, student, knowledge, attitude

## Abstract

**Background:** The misuse of antibiotics in dental practice significantly contributes to the escalation of antimicrobial resistance. This study aimed to assess the knowledge and attitudes of final-year dental students regarding perioperative antibiotic prophylaxis in oral surgery and to identify potential curricular improvements based on the findings. **Methods:** A questionnaire was administered to 117 final-year students at Gazi University Faculty of Dentistry in December 2024. The survey presented clinical scenarios related to common oral surgical procedures, evaluating participants’ antibiotic prescribing behaviors. Statistical analyses were performed using descriptive statistics and a One-Sample Chi-Square Test. **Results:** Students demonstrated a general tendency toward rational antibiotic use in routine clinical scenarios, with statistically significant response patterns favoring the avoidance of unnecessary prescriptions (*p* < 0.05). However, in complex or borderline cases such as impacted third molar extraction and dental implant placement, response variability was observed. Post hoc analyses revealed no statistically significant differences between closely distributed options, indicating inconsistencies in decision-making in more challenging scenarios. **Conclusions:** While final-year dental students exhibited a satisfactory level of knowledge regarding appropriate antibiotic use in standard surgical procedures, the variability observed in complex cases underscores the necessity for enhanced educational interventions. Incorporating updated, evidence-based antimicrobial stewardship principles and promoting clinical decision-making through case-based learning are essential to prepare future dental practitioners for responsible antibiotic prescribing, contributing to global efforts to mitigate antimicrobial resistance.

## 1. Introduction

Antibiotics are widely used in dentistry for the treatment of orofacial infections and, in specific cases, for prophylaxis to prevent postoperative complications based on defined clinical indications. Prophylactic antibiotics may be indicated in a limited number of high-risk cardiac conditions, such as specific congenital heart defects or previous infective endocarditis, according to current clinical guidelines. Infections in the oral and maxillofacial area can adversely affect soft and hard tissues and can rarely lead to life-threatening conditions. Common symptoms of infections in the oral and maxillofacial area include pain and swelling, and fever is less common. Fever is usually seen in cases where the infection has spread systemically. In the presence of clinical signs of infection, such as fever and swelling, antibiotics are prescribed for therapeutic purposes. In contrast, prophylactic antibiotics are occasionally considered before surgical procedures in medically compromised patients or high-risk interventions. To avoid risks and misuse, the appropriate antibiotic should be used in consideration of the optimal dosage for the appropriate duration of treatment and rational drug use [1,2].

Perioperative antibiotic prophylaxis, defined as the administration of antibiotics before or during surgery to prevent postoperative infection, remains a subject of debate in oral and maxillofacial surgery, with studies showing conflicting outcomes regarding its efficacy [3,4]. Some researchers classify this situation as primary prophylaxis, secondary prophylaxis, and eradication. Primary prophylaxis is the prevention of the initial infection, and secondary prophylaxis is the prevention of recurrence or the reactivation of a pre-existing infection. Eradication describes the elimination of a colonizing organism to prevent the development of infection. According to this classification, most antibiotic prophylaxis in dentistry consists of primary or secondary prophylaxis [5]. For this purpose, systemic or topical antibiotics are prescribed to reduce the risk of infection in the surgical field where tissue integrity is compromised. The prophylactic use of antibiotics against infections caused by susceptible microorganisms is common worldwide, and patients are usually given systemic antibiotics as a precaution [6]. In the literature, there are a number of studies evaluating the use of perioperative antibiotic prophylaxis in operations such as embedded third molar surgery, dental implant applications, the surgical treatment of osteoradionecrosis cases, and parotid gland surgery, which are frequently performed in the field of oral and maxillofacial surgery, trying to prevent unnecessary antibiotic use [3,7,8,9].

Despite the increasing awareness of antimicrobial resistance, several studies have shown persistent variability and inappropriate prescribing behaviors among dental professionals, including students. For instance, Lollobrigida et al. [5] and Sbricoli et al. [10] found that both practicing dentists and dental students demonstrated uncertainty regarding antibiotic prophylaxis in common oral surgical scenarios. These findings underscore the need to assess knowledge and attitudes specifically among final-year dental students to identify gaps and improve future curriculum design.

The growing threat of bacterial resistance due to inappropriate or excessive antibiotic use has become a global public health concern, particularly in the context of dental practices. So, this study aimed to evaluate the knowledge and attitudes of final-year dental students about antibiotic prophylaxis through a questionnaire to mitigate the increasing risk of antimicrobial resistance due to the increasing unconscious use of antibiotics in society and to obtain feedback through a questionnaire in terms of what changes should be made in the curriculum in light of these results. This study does not assume that current prescribing practices are appropriate or inappropriate but rather seeks to examine how final-year dental students apply their knowledge in clinical decision-making scenarios.

## 2. Results

The distribution of responses to all survey items is presented in Table 1. Statistical comparisons were conducted using One-Sample Chi-Square Tests, demonstrating that the differences in response frequencies across all questions were statistically significant (*p* < 0.05). In most clinical scenarios, respondents most frequently selected Option A, indicating a general tendency to refrain from unnecessary antibiotic use. These findings suggest a consistent pattern in students’ decision-making, reflecting adherence to rational prescribing principles in standard oral surgical procedures.

In questions where response frequencies were closely distributed between multiple options (e.g., Questions 7 and 10), post hoc pairwise comparisons were conducted using Bonferroni-adjusted Chi-Square Tests to determine whether the observed differences between specific response categories were statistically significant. In Question 7, although Options A (34.2%) and E (42.7%) were selected at similar rates, the difference between them did not reach statistical significance (*p* > 0.05). A similar pattern was observed in Question 10, where the preferences for Options B (50.4%) and E (49.6%) were nearly equal, and no statistically significant difference was detected between these options (*p* > 0.05). These findings suggest that final-year dental students in certain borderline clinical scenarios demonstrate variability in antibiotic prescribing decisions, possibly reflecting areas where further education or guideline clarification may be beneficial.

A significant *p*-value (*p* < 0.001) indicates that students did not respond randomly but showed a clear preference for specific antibiotic strategies.

**Figure 1:**
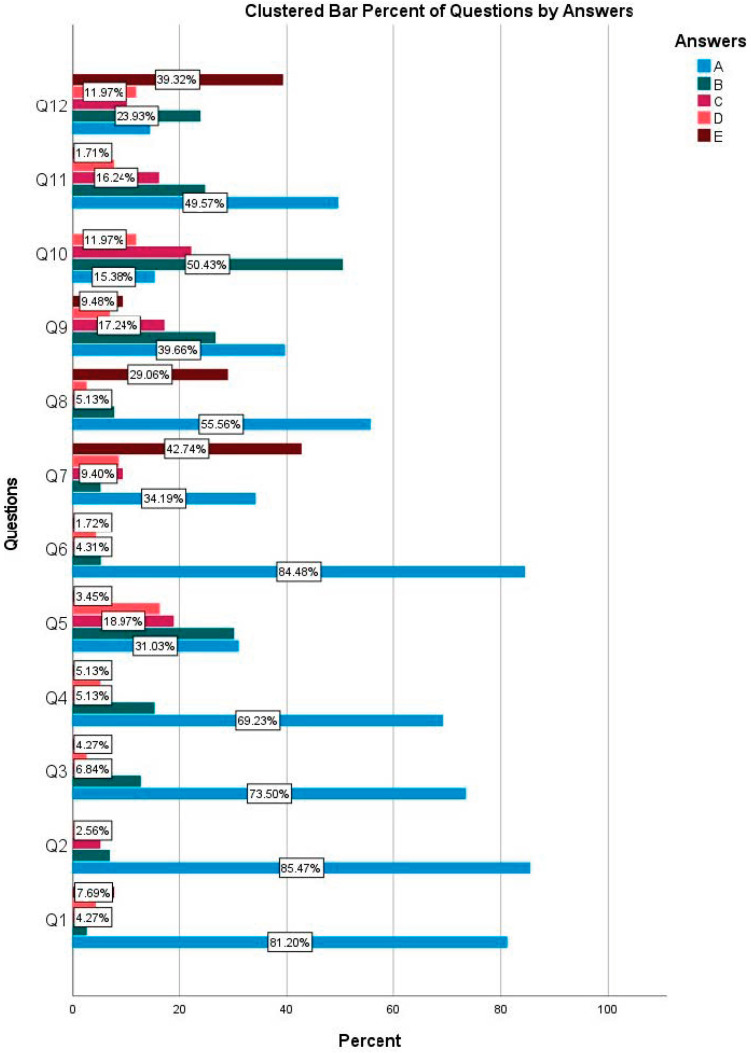
Bar plot of distribution of answers to questions.

Overall, students showed a consistent inclination toward avoiding unnecessary antibiotic prescriptions, especially in cases without systemic risk factors. However, in complex clinical scenarios, such as impacted third molar extraction and implant placement, substantial variability was observed, suggesting uncertainty or a lack of adherence to guidelines. These patterns are detailed in Table 1 and further illustrated in bar charts (Figure 1).

## 3. Discussion

To prevent systemic and local infections, dentists recommend taking antibiotics before and/or after surgery. Penicillins, Cephalosporins, Tetracyclines, and metronidazole are commonly used antibiotics in dentistry. Amoxicillin has always been the antibiotic of choice in dentistry because it is safe, bactericidal, and highly effective. It is administered orally at 500 mg every 8 h or 1000 mg every 12 h. Blood levels peak 1–2 h after oral administration. Amoxicillin also has some common side effects, such as diarrhea, nausea, vomiting, and abdominal discomfort. According to a study published in 2016 with 261 dentists, 216 dentists (82.7%) found it necessary to prescribe antibiotics for lower third molar surgery. Of these, 126 (58.3%) prescribed amoxicillin, and 74 (34.5%) prescribed amoxicillin + clavulanic acid, while 129 dentists (59%) found it appropriate to prescribe antibiotics both before and after surgery, and 10 (4.6%) stated that they would prescribe antibiotics only after surgery [11]. The controversy regarding the use of amoxicillin before and after surgery is due to differences in the results of studies. Therefore, this study aimed to investigate the knowledge and attitudes of final-year dental students regarding antibiotic use before or after various oral surgical procedures.

Antibiotic resistance, which occurs as a result of the inappropriate use of antibiotics, is considered a severe public problem worldwide, according to the Centers for Disease Control and Prevention reports. It is reported that 30% of prescribed antibiotics are unnecessary [12]. Moreover, according to a study on the use of antibiotics in dentistry, the use of antibiotics in simple tooth extractions does not significantly affect the occurrence of postoperative complications. Still, on the other hand, it may increase the risk of side effects of the drug [13,14,15]. Antibiotic prophylaxis should be performed in patients with immunosuppression, untreated congenital heart disease, or bacterial endocarditis history. In a study of 418 patients undergoing non-surgical tooth extraction, 280 patients were given antibiotics, while 138 patients were not given antibiotics, and the effects of antibiotics on the development of alveolitis after tooth extraction were evaluated. The researchers concluded that using antibiotics after tooth extraction was not beneficial in preventing alveolitis. In another study by Lollobrigida et al. [5], dentists mainly selected the option that patients should not receive antibiotic treatment in all questions regarding administering antibiotics in erupted tooth extractions. In this study, the first six questions of the questionnaire were related to the extraction of erupted teeth in individuals with various disease scenarios. Similarly, the results significantly indicated that antibiotics should not be used (*p* < 0.05). Only in Question 5 did the participants respond with the second highest rate (30.2%) that antibiotics should be given one hour before the procedure. The extraction of the third molars in different positions is a common practice in oral and maxillofacial surgery. Although third molar extraction is generally an easy procedure, some complications may be encountered during or after surgery. According to a study by Blondeau and Daniel [16], the frequency of postoperative complications, including alveolitis, infection, and paresthesia of the inferior alveolar nerve, was reported as 6.9%. In a more recent study by Candotto et al. [17], the frequency of postoperative complications, including alveolitis (0.5% to 32.5%), infection (0.9% to 4.2%), postoperative bleeding (0.2% to 1.5%), and transient and permanent dysfunction of the inferior alveolar nerve (0.6% to 5.5%, 0.1% to 0.9%), was summarized. These complications may lead to the deterioration of the patient’s oral and systemic functions and decreased quality of life during or after surgery. Although complications such as infection and alveolitis may occur after third molar extraction, the effectiveness of prophylactic antibiotics in preventing these outcomes remains controversial. Several systematic reviews have questioned the routine use of antibiotics in these cases, highlighting the need for a patient-specific, evidence-based approach [4,7]. In this context, prophylactic antibiotic administration may also be considered. In this study, the seventh question of the survey given to the students was about using antibiotics to completely extract impacted wisdom teeth. In the study conducted by Lollobrigida et al. [5], only 7.7% of the participants stated that they would not prescribe antibiotics or would prescribe a single preoperative dose for the surgical extraction of impacted wisdom teeth (14.8%), whereas 77.4% of the participants stated that they would prescribe pre- and postoperative antibiotics or only postoperative antibiotics. According to a study conducted by Sbricoli et al. [10] with 298 dentist participants, the % of those who reported that they would not prescribe antibiotics for routine impacted third molar extraction was 18%. According to the results of our study, the percentage of those who found it appropriate to apply broad-spectrum antibiotics for 5–7 days after the procedure was 42.7%. This was followed by the option of not applying antibiotic therapy, at 34.2%.

Today, dental implant treatment is one of the most effective methods for restoring both function and esthetics to patients. However, as with any surgical procedure, there is a possibility of complications when applying dental implants. For instance, the most common risk is infection, typically detected in the peri-implant tissues. Unfortunately, dentists and patients must deal with various challenges due to peri-implant diseases when an infection develops. Additionally, early failure in dental implant applications can be defined as implant loss due to a lack of osseointegration within the first few months after implant placement. This type of failure, often associated with bacterial contamination during implant surgery, can also be influenced by other factors such as surgical technique, implant characteristics (including height, length, and surface features), surgeon experience, periodontitis history, and smoking habits. To reduce the risk of infection leading to early implant loss due to insufficient osseointegration, antibiotics may be prescribed to the patient along with implant surgery [3,18]. In this study, the eighth question of the questionnaire administered to students was related to the use of antibiotics in the placement of intraosseous dental implants. According to a survey by Rodríguez Sánchez et al. [19], approximately 84% of the dentists who participated in the survey routinely prescribed prophylactic antibiotics along with oral implant surgery, 15.6% prescribed antibiotics only in specific situations, and only one did not prescribe any antibiotics at all. In a study conducted by Sbricoli et al. [10], 18% of respondents stated they would not prescribe antibiotics for routine dental implant placement. Additionally, in a survey by Lollobrigida et al. [5], 20.1% of participants indicated that they prescribed only a single dose of antibiotics before surgery for patients undergoing routine implant surgery. In comparison, 14.8% reported that they never prescribed antibiotics. In our study, 55.6% of the students participating in the survey indicated that they would not prescribe antibiotics for routine implant surgery, followed by the option “a broad-spectrum antibiotic is applied for 5–7 days postoperatively” at 29.1%.

Diabetes mellitus (DM) is a systemic disease affecting a large population worldwide and affects millions of individuals. It is a metabolic disorder characterized by inadequate insulin secretion from the pancreas and abnormal insulin levels, causing microvascular complications and hyperglycemia. Hyperlipidemia, obesity, and impaired healing are frequently observed in patients with DM. Tooth loss, delayed wound healing, xerostomia, dental caries, burning mouth syndrome, lichen planus, and even osteomyelitis of the jaw bones are common in patients with DM. This condition can complicate the treatment of these patients and jeopardize the outcomes of various oral diseases [20]. There is a lot of evidence supporting these findings in the literature. Patients with diabetes are at higher risk of infection or delayed wound healing after surgical operations [21]. Many studies in the literature recommend antibiotic prophylaxis as an appropriate preventive measure for diabetic patients due to the possibility of delayed wound healing and infection after surgical procedures are performed in the oral cavity. Current recommendations for antibiotic prophylaxis before surgical procedures are not definitive and are based on expert opinions [22]. Therefore, the ninth question of the survey administered in this study is related to the surgical extraction of a tooth planned to be removed in a diabetic patient. Lollobrigida et al. [5] reported that 26% of dentists preferred not to prescribe antibiotics when performing a non-surgical tooth extraction on a diabetic patient. According to our study, 39.7% of the participants considered antibiotics unnecessary in such cases. The findings of both studies are consistent with each other.

The third molars often erupt partially or remain impacted due to genetics or the reduced use of the jaws. The incidence of impacted teeth ranges from 16.7% to 68.6%, and pericoronitis is observed in 4.92% of young individuals between 20 and 25 years of age. Pericoronitis can spread to surrounding tissues and the lymphatic system, leading to complications such as abscesses, buccal fistulas, and osteomyelitis [23]. Antibiotic administration is recommended for severe cases of pericoronitis to prevent the spread of infection [24]. Therefore, the 10th question of the questionnaire administered in the present study is related to the extraction of a partially impacted tooth in a patient with early pericoronitis, and the results showed that 50.4% of survey respondents preferred to re-evaluate the patient 3 days after using local antiseptic and anti-inflammatory drugs and avoid using antibiotics in the first place, while 49.6% preferred to perform immediate surgical extraction and prescribe broad-spectrum antibiotics for 5 days after the procedure. In another study, the authors determined that participants preferred the same approach by 79.3%.

A periodontal abscess is defined as a lesion that develops over time, resulting in significant periodontal destruction and clinical symptoms typically indicated by localized pus accumulation. The treatment of a periodontal abscess is initiated once the acute condition is managed. If the tooth and surrounding tissues are severely damaged and the prognosis is poor, tooth extraction is considered the most effective treatment option [25]. In cases where tooth extraction is unnecessary, foreign bodies are initially removed through debridement. Next, irrigation is performed in the periodontal pocket, and the condition is monitored. The 11th question of the questionnaire used in this study pertains to periodontal abscess. This study found that 49.6% of participants preferred performing abscess drainage from the pocket using manual and ultrasonic root planning without an antibiotic prescription. In contrast, another study indicated that most participants preferred a postoperative course of 5 days of antibiotic treatment, followed by manual or ultrasonic debridement in such cases [5].

Medication-related osteonecrosis of the jaw (MRONJ) is defined as exposed bone in the maxillofacial region that fails to heal within 8 weeks. It occurs in patients with a history of using bone-modifying agents and who have not undergone head and neck radiation [26]. This condition may also be associated with intraoral or extraoral fistulas. Systemic antibiotics are typically prescribed perioperatively for tooth extractions. Consequently, question 12 of the questionnaire in this study focuses on managing standard tooth extraction in patients receiving bisphosphonate therapy. The results indicated that 39.3% of participants deemed it appropriate to refer the patient to an oral surgeon. In contrast, participants in a similar study preferred using perioperative antibiotic prophylaxis for 2 to 3 days for non-surgical tooth extraction in MRONJ patients, with 55% opting for this approach.

Previous large-scale reviews have demonstrated a clear relationship between community-level antibiotic consumption and the emergence of antimicrobial resistance. For instance, a meta-analysis by Bell et al. [27] evaluating 243 eligible studies found a significant association between antibiotic use and resistance, with a pooled odds ratio of 2.3 (95% CI: 2.2–2.5), suggesting that higher antibiotic consumption significantly increases the likelihood of resistant bacterial strains. Interestingly, their meta-regression analysis revealed regional variations, with southern European countries showing a stronger correlation between consumption and resistance. These findings reinforce the importance of minimizing unnecessary antibiotic prescriptions, not only to prevent individual adverse effects but also to mitigate broader public health consequences at the community and regional levels. Our study’s observation of inconsistent prescribing behaviors among final-year dental students in complex cases echoes the broader issue of knowledge gaps that may contribute to excessive or inappropriate antibiotic use. Integrating robust antimicrobial stewardship principles into dental education is therefore essential to curb resistance trends consistent with those reported in this large-scale meta-analytic study.

In addition to community-level associations between antibiotic consumption and resistance, individual-level antibiotic exposure has also been shown to significantly increase the risk of developing resistant infections. A comprehensive systematic review and meta-analysis of 24 studies by Costelloe et al. [28] demonstrated that patients prescribed antibiotics in primary care settings—particularly for respiratory or urinary tract infections—had markedly higher odds of harboring resistant bacteria, with pooled odds ratios of 2.5 (95% CI: 2.1–2.9) for urinary pathogens and 2.4 (95% CI: 1.3–4.5) for respiratory pathogens within two months of treatment. Although resistance gradually decreased over time, it remained elevated for up to 12 months. Notably, repeated or prolonged antibiotic courses were associated with even higher resistance rates. These findings underscore the long-term implications of inappropriate antibiotic prescribing at the individual level, further supporting the need for targeted educational strategies among dental students. Our study’s identification of variable prescribing behavior in complex scenarios emphasizes the importance of equipping future practitioners with the knowledge to minimize unnecessary antibiotic exposure and its downstream effects.

## 4. Materials and Methods

This study employed a descriptive, cross-sectional survey design to assess the knowledge and attitudes of final-year dental students regarding perioperative antibiotic prophylaxis.

The survey used by Lollobrigida et al. [5] in a study published in 2021 was administered to final-year students of Gazi University Faculty of Dentistry (Table 2). The students who answered the survey already took courses on perioperative antibiotic prophylaxis during their 4th year of education and answered the survey in December 2024 after passing these courses. A total of 117 students gave their informed consent and participated in the survey. The first page of the survey included general information provided about the survey and explained the purpose of this research. Participants were informed about their right not to participate in the survey, and everyone volunteered to participate.

The questionnaire developed by Lollobrigida et al. [5] (2021) was translated into Turkish using a forward–backward translation method by two independent bilingual experts. While the questionnaire was not subjected to full psychometric validation for the Turkish population, the content was reviewed by specialists in oral surgery to ensure cultural and conceptual equivalence.

The survey was administered in paper format during scheduled classroom sessions in December 2024 under researcher supervision.

In the survey (Table 2), surgical procedures commonly performed by oral and maxillofacial surgeons were presented with different clinical scenarios, and the participants were asked to choose the most appropriate option or treatment regimen in terms of perioperative antibiotic prophylaxis from the options. Single or multiple non-surgical extraction cases were suggested in Questions 1, 2, 3, 4, 5, 6, 9, and 12. Questions 7 and 8 described the surgical extraction of an impacted wisdom tooth and the application of a single dental implant, respectively. Questions 10 and 11 described the pericoronitis and periodontal abscess cases without systemic signs and symptoms, respectively. Question 12 presented the treatment options for a non-surgical extraction case in an osteoporosis patient receiving regular oral bisphosphonate treatment. The clinical variables in the questions were determined as the age of the patients, systemic conditions, and the type/number of surgical procedures.

### 4.1. Power Analysis

In this study, the sample size was calculated at a 95% confidence level, assuming that the population size is known. The formula for sample size estimation for finite populations was used for the calculation [29]. The population size was determined to be 130, and the expected proportion of the sample profile of interest was assumed to be 0.50. When the significance level was set at α = 0.05, the corresponding z-score was 1.96. Based on these parameters, the minimum required sample size was calculated as 98.

### 4.2. Statistical Analysis

In this study, descriptive statistics (number and percentage) of the data are provided. The Chi-Square Test for One Sample was used to test the differences between the rates of responses given to categorical variables. Analyses were performed in the IBM SPSS 25 program.

The primary hypothesis of this study was that the observed frequencies significantly differ from the expected frequencies; accordingly, a One-Sample Chi-Square Test was used to determine whether the distribution of responses to each survey item significantly deviated from an equal probability model, indicating a preferred decision pattern among students. A significance level of *p* < 0.05 was applied.

### 4.3. Ethical Approval

The study protocol was ethically approved by the Ethics Committee of Gazi University at its meeting on 9 October 2024, with the research code number 2024-1526. Informed voluntary consent forms were obtained from all individuals participating in this study.

All methods were performed in accordance with relevant guidelines and regulations.

## 5. Conclusions

In conclusion, in many countries, the use of antibiotics in dental treatments has become increasingly common in routine clinical practice. Dentists prescribe antibiotics prophylactically to protect their patients from infections. Often, this antibiotic prescription is unnecessary, leading to the development of antibiotic resistance in the community and negatively impacting the country’s economy. Therefore, it is of great importance to teach dental students the correct use of antibiotics under the concept of rational drug use, and such survey studies provide valuable insights into evaluating the knowledge and attitudes of young dental candidates. This study reveals that final-year dental students possess a general awareness of appropriate antibiotic use, particularly in straightforward oral surgical procedures. Nonetheless, their inconsistent decision-making in borderline or medically complex scenarios underscores a need for more robust and clinically integrated instruction on antimicrobial stewardship. Incorporating updated evidence-based guidelines and reinforcing clinical reasoning through practical case-based learning may better equip future dental professionals to make informed, judicious decisions regarding antibiotic prescription. This approach is essential not only for improving patient outcomes but also for combating the growing threat of antibiotic resistance in dental and broader healthcare settings.

The findings of this study indicate that while students generally demonstrate rational antibiotic use in standard scenarios, notable inconsistencies arise in complex clinical contexts. These results suggest targeted educational improvements are needed in undergraduate dental training to strengthen clinical decision-making aligned with evidence-based prophylaxis guidelines.

Our findings highlight inconsistencies in prescribing patterns that reflect a need for stronger alignment between dental education and current evidence-based guidelines rather than reinforcing traditional prescribing habits.

## Figures and Tables

**Table 1:** Distribution of answers to questions.

	A		B		C		D		E			
	n	%	n	%	n	%	n	%	n	%	Test Statistics	*p*-Value
Q1	**95**	**81.2**	3	2.6	5	4.3	5	4.3	9	7.7	274.667	<0.001 *
Q2	**100**	**85.5**	8	6.8	6	5.1	3	2.6	0	0.0	228.607	<0.001 *
Q3	**86**	**73.5**	15	12.8	8	6.8	3	2.6	5	4.3	212.872	<0.001 *
Q4	**81**	**69.2**	18	15.4	6	5.1	6	5.1	6	5.1	181.846	<0.001 *
Q5	**36**	**31.0**	35	30.2	22	19.0	19	16.4	4	3.4	29.776	<0.001 *
Q6	**98**	**84.5**	6	5.2	5	4.3	5	4.3	2	1.7	301.845	<0.001 *
Q7	40	34.2	6	5.1	11	9.4	10	8.5	**50**	**42.7**	69.197	<0.001 *
Q8	**65**	**55.6**	9	7.7	6	5.1	3	2.6	34	29.1	118.342	<0.001 *
Q9	**46**	**39.7**	31	26.7	20	17.2	8	6.9	11	9.5	41.845	<0.001 *
Q10	18	15.4	**59**	**50.4**	26	22.2	14	12.0	58	49.6	42.897	<0.001 *
Q11	**58**	**49.6**	29	24.8	19	16.2	9	7.7	2	1.7	81.761	<0.001 *
Q12	17	14.5	28	23.9	12	10.3	14	12.0	**46**	**39.3**	33.812	<0.001 *

* *p* < 0.05, higher rates are in bold.

**Table 2:** Questionnaire form.

Q1—Non-surgical extraction of tooth 36 in infraocclusion with odontomy in an 11-year-old patient with no history of systemic disease: (a) No antibiotic therapy is applied (b) One dose of broad-spectrum antibiotic is applied 1 h before the procedure (c) Broad-spectrum antibiotic is applied 1 h before the procedure and continued for up to 5 days after the procedure (d) Broad-spectrum antibiotic is applied starting 24–48 h before surgery and continued for 3–4 days after the procedure (e) Broad-spectrum antibiotic is applied for 5–7 days after the procedure Q2—Non-surgical extraction of tooth 24 with extensive caries and no acute or chronic inflammatory lesions in a 56-year-old patient with controlled arterial hypertension, (a) No antibiotic therapy is applied (b) One dose of broad-spectrum antibiotic is applied 1 h before the procedure (c) Broad-spectrum antibiotic is applied 1 h before the procedure and up to 5 days after the procedure (d) Broad-spectrum antibiotic is applied starting 24/48 h before the surgery and up to 5 days after the procedure Broad spectrum antibiotics are applied for 3–4 days (e) Broad spectrum antibiotics are applied for 5–7 days after the procedure Q3—Non-surgical extraction of tooth number 26 with grade 3 mobility in an 88-year-old ASA II patient who is on anticoagulants and has controlled hypertension., (a) No antibiotic therapy is applied (b) One dose of broad spectrum antibiotics are applied 1 h before the procedure (c) Broad spectrum antibiotics are applied 1 h before the procedure and up to 5 days after the procedure (d) Broad spectrum antibiotics are applied 24/48 h before the surgery and for 3–4 days after the procedure (e) Broad spectrum antibiotics are applied for 5–7 days after the procedure Q4—Non-surgical extraction of teeth numbers 34 and 35 in a 75-year-old patient with controlled arterial hypertension and well-controlled type II diabetes, without acute or chronic inflammatory lesions, (a) No antibiotic therapy is applied (b) One dose of broad-spectrum antibiotics are applied 1 h before the procedure (c) Broad spectrum antibiotics are applied 1 h before the procedure and up to 5 days after the procedure (d) Broad spectrum antibiotics are applied starting 24/48 h before the surgery and for 3–4 days after the procedure (e) Broad spectrum antibiotics are applied for 5–7 days after the procedure Q5—A 57-year-old patient with a history of myocardial infarction, high cholesterol, anticoagulant medication, and controlled hypertension presents with three root remnants in a single-rooted tooth. Periapical granuloma was detected in two of the roots on the X-ray. For non-surgical extraction of this tooth, (a) No antibiotic therapy (b) One dose of broad-spectrum antibiotic is applied 1 h before the procedure (c) Broad-spectrum antibiotic is applied 1 h before the procedure and up to 5 days after the procedure (d) Broad-spectrum antibiotic is applied starting 24/48 h before the surgery and up to 3–4 days after the procedure (e) Broad-spectrum antibiotic is applied for 5–7 days after the procedure Q6—Non-surgical extraction of five root remnants (teeth 13, 15, 22, 24, and 26) without acute or chronic periapical lesions in a 68-year-old patient with no history of systemic disease, (a) No antibiotic therapy (b) One dose of broad-spectrum antibiotic is applied 1 h before the procedure (c) Broad-spectrum antibiotic is applied 1 h before the procedure and up to 5 days after the procedure (d) Broad-spectrum antibiotic is applied starting 24/48 h before the surgery and up to 3–4 days after the procedure (e) Broad spectrum antibiotics are applied for 5–7 days after the procedure Q7—Surgical extraction of tooth number 48, which is completely impacted in mandible, in a 25-year-old patient with no systemic disease, (a) No antibiotic therapy is applied (b) One dose of broad-spectrum antibiotic is applied 1 h before the procedure (c) Broad-spectrum antibiotic is applied 1 h before the procedure and up to 5 days after the procedure (d) Broad-spectrum antibiotic is applied starting 24/48 h before the surgery and for 3–4 days after the procedure (e) Broad-spectrum antibiotic is applied for 5–7 days after the procedure	Q8—Placement of dental implants in the mandibular molar region by raising a mucoperiosteal flap in a 54-year-old patient with no systemic disease, (a) No antibiotic therapy is applied (b) A dose of broad-spectrum antibiotic is applied 1 h before the procedure (c) Broad-spectrum antibiotic is applied 1 h before the procedure and up to 5 days after the procedure (d) Broad-spectrum antibiotic is applied starting 24/48 h before surgery and for 3–4 days after the procedure (e) Broad-spectrum antibiotic is applied for 5–7 days after the procedure Q9—Non-surgical extraction of teeth numbers 14 and 15 in a 55-year-old patient with metabolic syndrome and no acute or chronic periapical lesions (patient’s fasting blood sugar: 160 mg/dL) (a) No antibiotic therapy is applied (b) A dose of broad-spectrum antibiotic is applied 1 h before the procedure (c) Broad-spectrum antibiotic is applied 1 h before the procedure and up to 5 days after the procedure (d) 24/48 h before and 3–4 days after the procedure broad-spectrum antibiotics are applied (e) Broad-spectrum antibiotics are applied for 5–7 days after the procedure Q10—A 17-year-old patient with mild pain in tooth number 38, which has partial bone retention and has been ongoing for 2 days. The patient has no fever or purulent exudate in the pericoronal tissue on clinical examination, but the tissue was found to be erythematous and edematous. In this patient, (a) Broad-spectrum antibiotics and anti-inflammatory/analgesic drugs are prescribed for 5 days, (b) Local antiseptics (chlorhexidine), anti-inflammatory/analgesic drugs and oral hygiene instructions; If there is no regression in symptoms in 3 days, broad-spectrum antibiotics are prescribed for 5 days, (c) Spontaneous regression in symptoms is expected; If there is no regression in symptoms within 3 days, broad-spectrum antibiotics are prescribed for 5 days, (d) Immediate surgical extraction is performed, and broad-spectrum antibiotics are prescribed for 5 days after the procedure. Q11—A 54-year-old male patient presents with a 7 mm diameter fluctuant swelling in the buccal mucosa adjacent to tooth number 26. The tooth is not luxated. Patient has no fever or lymphadenopathy, palpation revealed suppuration in the sulcus, and probing in three different areas of the vestibule revealed pocket depths of 9 mm, 8 mm, and 9 mm. In this patient, (a) Manual and ultrasonic root planing, along with abscess drainage from the pocket, are performed. (b) After manual and ultrasonic root planing and abscess drainage from the pocket, 5 days of broad-spectrum antibiotics are prescribed. (c) Manual and ultrasonic root planing are performed, followed by 5 days of broad-spectrum antibiotics. (d) Following a single dose of antibiotic prophylaxis 1 h before the procedure, the tooth is extracted. (e) Other Q12—A 62-year-old patient who has been using bisphosphonates (70 mg oral sodium alendronate once a week) for 11 years due to osteoporosis has had his treatment interrupted for 30 days. Patient has no other systemic diseases. For non-surgical extraction of the root of this patient’s cervically fractured tooth number 35, (a) A single dose of broad-spectrum antibiotic is used 1 h before surgery and local antiseptic and mouthwash (0.2% chlorhexidine) before extraction (b) Local antiseptic (0.2% chlorhexidine); use of antibiotic therapy (amoxicillin + metronidazole) drugs for 2–3 days before surgery and 5–7 days after surgery (c) Use of local antiseptics (0.2% chlorhexidine); use of antibiotic (amoxicillin + metronidazole) for 5–7 days after surgery (d) Use of local antiseptics (0.2% chlorhexidine); use of antibiotics (amoxicillin + metronidazole) starting 1 day before surgery and continuing until 5–7 days after surgery (e) I refer the patient to an oral surgeon

## Data Availability

The data presented in this study are available on request from the corresponding author due to participant confidentiality and ethical considerations.
